# Layer Transfer from Chemically Etched 150 mm Porous Si Substrates

**DOI:** 10.3390/ma4050941

**Published:** 2011-05-23

**Authors:** Barbara Terheiden, Jan Hensen, Andreas Wolf, Renate Horbelt, Heiko Plagwitz, Rolf Brendel

**Affiliations:** Institut für Solarenergie Forschung Hameln (ISFH), Am Ohrberg 1, 31860 Emmerthal, Germany; E-Mails: j.hensen@isfh.de (J.H.); a.wolf@isfh.de (A.W.); r.horbelt@isfh.de (R.H.); h.plagwitz@isfh.de (H.P.); r.brendel@isfh.de (R.B.)

**Keywords:** layer transfer process, porous silicon, stain etching

## Abstract

We demonstrate for the first time the successful layer transfer of an epitaxially grown monocrystalline Si film from a purely chemically etched porous Si substrate of 150 mm diameter to a glass carrier. The surface conditioning for all Si layer transfer processes based on porous Si has been, up to now without exception, carried out by electrochemical etching. In contrast, our chemical stain etching process uses an aqueous HF-rich HF/HNO_3_ solution. The porosity increases with increasing doping concentration of the Si substrate wafer and with increasing porous layer thickness. In contrast to the electrochemically etched double layers, the porosity profile of the stain etched substrates is highest at the original wafer surface and lowest at the interface between the porous layer and the Si bulk. The epitaxy process is adapted to the high porosity at the surface with regard to the reorganization of the porous layer.

## 1. Introduction

Electrochemically etched porous silicon double layers are commonly applied for Si layer transfer processes [1]. These double layers consist of a thin layer with a high porosity (~50%) beneath a thicker layer of lower porosity (~20%) [2]. The layer of high porosity serves as a predetermined breaking point for the later transfer. The low porosity layer acts as a seed layer for the following epitaxy process. During the epitaxy process the porous layers reorganize [3] driven by the minimization of the surface energy [4]. This leads to a closure of the surface and the formation of a thin Si layer [5] as well as to the dissolution of the high porosity layer leaving just a few Si bridges which connect the substrate wafer with the epitaxial Si layer.

Up to now this PSI (porous silicon) process [6] has been based on an electrochemically etched porous Si double layer system consisting of a 1 µm-thick low porosity layer on top of a 0.2 µm-thick high porosity layer. A sharp interface separates the two layers. An increase of the etching current causes the change in porosity. An etching cell filled with aqueous HF as electrolyte and the silicon wafer acting as the anode is used for the electrochemical etching.

Although good solar cell results were achieved by layer transfer techniques based on electrochemical etching, the throughput of the porosification process is low because each wafer has to be electrically contacted. Up to now the homogeneous anodization of batches of wafers has not been shown.

Therefore the transfer of the PSI-process into an industrial production environment requires a high throughput approach for the formation of the porous Si layer that overcomes the challenge of electrically contacting each wafer and that guarantees a uniform porosity of the layers on the entire area of the wafer. The formation of porous Si layers by purely chemical stain etching [7,8] avoids the necessity of electrical contacts and allows etching a whole batch of wafers at once. Stain etching, *i.e*., etching of Si by an aqueous solution of HF/HNO_3_, has been investigated for about 50 years for different applications [9,10]. However, its application in a layer transfer process like the PSI process has not been demonstrated up to now.

The main challenge of applying the stain etching process for porous Si layer formation in the PSI process, is to form a porous layer with a porosity profile that is suitable for both, the epitaxial growth of high-quality monocrystalline silicon, and its subsequent lift-off from the substrate. Furthermore, a sufficiently high uniformity of the porous silicon properties has to be ensured for layer transfer of large areas like wafers of 150 mm diameter.

The main difference between the electrochemical etching and the stain etching process is that the already porosified Si is attacked within the latter process. This results in a porosity profile with a continuous gradient that is opposite to the one generated electrochemically for layer transfer purposes, *i.e*., at the interface Si bulk/porous Si the lowest porosity is found while the highest porosity is located at the original wafer surface. In case of electrochemical etching, the original wafer surface is preserved regardless of the etching depth. As stain etching proceeds into the wafer bulk, the porosity at the original wafer surface increases until the original surface is dissolved. That means that for each substrate a certain maximum porous layer thickness is given, at which the wafer surface is still intact and therefore suitable for epitaxy. This maximum porous layer thickness depends mainly on the doping type and dopant concentration of the substrate wafer, as well as on the concentration of HNO_3_ and HF in the etching solution.

Turner, and later with more details Shih, considered the stain etching of silicon as an electrochemical process [8,11]. Nitric acid is reduced in a cathodic partial reaction and three holes are created which yield the anodic oxidation of silicon.

In the stain etching process, HNO_3_ plays the same role as the anodic current density in the electrochemical one. That means a HNO_3_-rich solution corresponds to the high-current regime in the electrochemical process and electropolishing takes place. But a HF-rich solution results in a process that is limited by the availability of holes and gives rise to porous Si formation *i.e*., the pits at the surface will be enlarged to form a pore. Within this regime, the porous layer formation rate rises with an increasing amount of HNO_3_.

Velasco [12] stated that the rate determining step of the whole corrosion process is the reduction of nitrate to NO. The redox couple to be considered is NO_3_^−^/NO. The difference of the corresponding redox potential (the pseudo Fermi level [13,14]) to the flat band potential of the p-type wafer is the driving force of electron transfer from Si surface atoms to the nitrate ions in solution. This electron transfer corresponds to a hole injection into the valence band.

Kolasinski [15] generated a comprehensive understanding of the processes taking place during chemical porous Si layer formation.

We demonstrate in this work for the first time that the pure chemical etching is successfully applied to generate a porous layer for subsequent lift-off of an epitaxially grown monocrystalline Si layer from a wafer with 150 mm diameter. The main topics we investigated are the dependence of the porosity profile on the silicon substrate doping concentration, the lift-off properties in dependence of the porous layer thickness, and the reorganization of the purely chemically etched porous layers at elevated temperatures in hydrogen atmosphere in the course of the epitaxy process.

## 2. Experimental

The porosity in the as etched state was measured as a function of the etching duration and of the doping density. The reorganization of the porous Si layer during the epitaxy process was also investigated in this work. We applied annealing steps to optimize the reorganization for the subsequent transfer process.

Highly boron-doped monocrystalline Si wafers of 150 mm and 10 mm in diameter with a resistivity between 1 and 12 mΩcm were subjected to the same temperature controlled aqueous HF-rich stain etching solution. The wafer and the solution were moved relatively to each other to avoid the sticking of gas bubbles on the wafer surface. The etching solution was fed with silicon before the first sample was immersed to guarantee that the etching process was reliable and thus reproducible [16], *i.e*., the same etching solution and etching set up were used for all etching experiments in this study.

The porous layer thickness of the etched samples was obtained by applying the appropriate etching duration. Determining the porous layer thickness for a given resistivity with a scanning electron microscope at a known etching duration and a linear extrapolation of this etch rate leads to the desired porous layer thickness. The etching depth was uniform on the entire wafer within ±6% at an etching depth of 1.4 µm.

Within the epitaxy process in hydrogen atmosphere an only 500 nm thick Si layer with a boron doping concentration of 3 × 10^16^ cm^−3^ was deposited with a subsequent annealing step at 1,100 °C of 40 min simulating the epitaxy of a 30 µm thick Si layer.

The epitaxy of a thicker Si layer was omitted to allow the preparation of accurate cross sections of the porous layers for the characterization by scanning electron microscopy (SEM). Thicker epitaxial Si layers can make the porous Si layer to lift-off at the cleaved edge, which leads to non planar cross sections, because the porous starting layer might be removed from the cross section area.

The porosity was determined applying gravimetrical measurements. The porosity follows from weighing the sample before (m_1_) and after the porosification (m_2_) to determine the amount of Si removed during etching. A potassium bath selectively removed the porous silicon and weighing gave the mass of the remaining substrate (m_3_). The porosity then was calculated from:

[P = (m_1_ − m_2_)/(m_1_ − m_3_)] (1)

## 3. Results

The etched layers were mesoporous in all cases as expected from electrochemical etching in HF-based solutions. The pore shape was also similar to the electrochemically etched pores. The pores had a diameter of about 10 nm. The main pores from which the branches grew were spaced between 50 and 100 nm. The surface of the porous Si layer was, in all cases shown here, the original wafer surface. The color of the wafer surface indicates the uniformity of the layer thickness over the entire wafer for specific resistivities of (1.3…2.5) mΩcm, where the porosity, and therefore the change of the refractive index, is large enough. The colors result from interferences between the reflections of light at the air/porous Si interface and the porous Si/Si-wafer interface. Since, for a specific resistivity of 2.2 mΩcm, one cycle from violet to violet (next refraction order) corresponded to 90 nm layer thickness, a thickness variation of about 10 nm is easily detected by the human eye. The color impression became non-uniform with dark spots when the original surface started to get etched away *i.e*., the porosity became 100%.

### 3.1. Porosity versus Doping Concentration

The porosity of p-type Si wafers with resistivities of 1.3, 2.5, 6, 7.7 and 11.2 mΩcm was determined at a porous layer thickness of about 600 nm for all samples.

**Figure 1:**
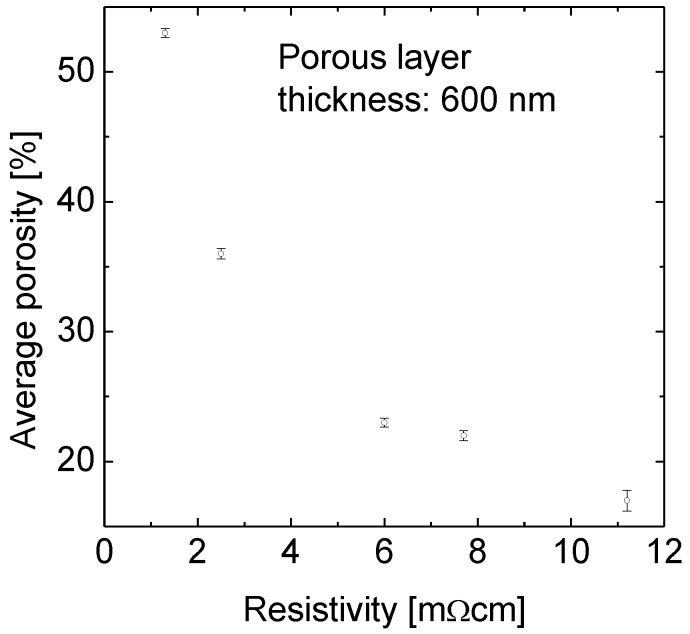
The average porosity as a function of the resistivity of the Si substrate wafer. The p-type doped wafers with resistivities of 1.3, 2.5, 6, 7.7 and 11.2 mΩcm were immersed in the temperature controlled aqueous HF-rich stain etching solution. The etching solution was fed with silicon before the first sample was immersed. There is a strong increase in porosity with decreasing resistivity.

Figure 1 demonstrates the strong impact of the substrate doping on the porosity. The porosity varies between 17% for the 11.2 mΩcm sample and 56% for the 1.3 mΩcm one. That means small variations in resistivity lead to significant changes in porosity.

### 3.2. Porosity *versus* Porous Layer Thickness

A set of 14 pieces of silicon wafers (100 mm diameter) with a resistivity of 2.2 mΩcm was divided in seven pairs. We increased the porous layer thickness by about 200 nm, from 200 nm up to 1,400 nm from pair to pair. Even at the maximum etching duration the surface of the porous layer was found to be still at the original wafer surface. Hence the porosity at the surface was below 100%.

One wafer of each pair was then examined as etched, while the other one underwent the epitaxy process. For the as etched wafers the average porosity was determined as a function of the porous Si layer thickness *i.e*., of the etching duration.

**Figure 2:**
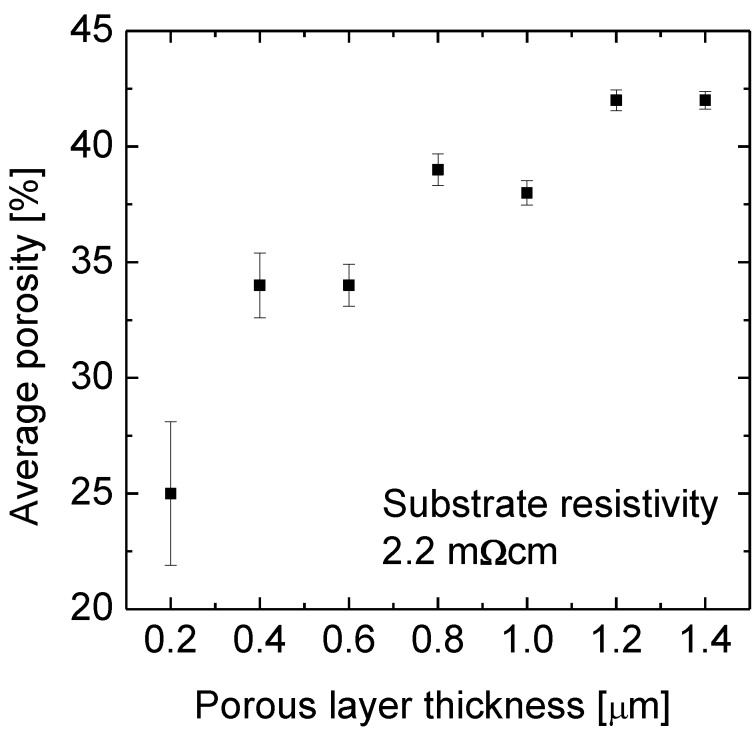
The average porosity as a function of the etching depth into a Si wafer with a resistivity of 2.2 mΩcm. The wafers were etched in the temperature controlled aqueous HF-rich stain etching solution. The etching solution was fed with silicon before the first sample was immersed. The average porosity increases from 25% at a layer thickness of 0.2 µm to 42% at 1.4 µm.

Figure 2 shows the average porosity as a function of the layer thickness. The porosity is only 25% for the 200 nm thick layer and 42% for the 1.4 µm thick porous Si layer. This difference is the lower limit for the porosity gradient in the 1.4 µm thick layer. The freshly etched part has a porosity of maximum 25% as known from the 200 nm thin sample while the effective porosity of 42% indicates that the maximum porosity lies above 42%. A linear porosity gradient would mean a porosity of 59% at the original wafer surface. That means the chemical etching provides a porosity profile exactly opposite to the one generated electrochemically.

We find that the etch rate does not change with layer thickness. From one sample that is etched to a medium layer thickness of 450 nm we determined R = 37 nm/min.

**Figure 3:**
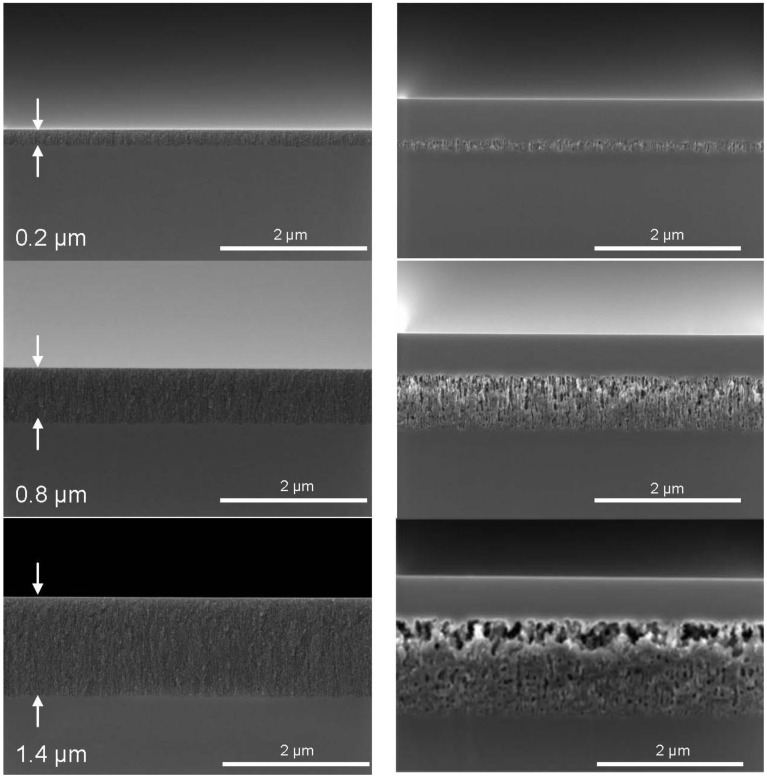
Evolution of the porous layer morphology with increasing thickness. A set of 14 pieces of silicon wafers (100 mm diameter) with a resistivity of 2.2 mΩcm was divided in seven pairs. The porous layer thickness was increased by applying the respective etch duration. Left: before the epitaxy process, right: after the epitaxy process. The porosity gradient is not visible in the as etched state but becomes obvious after the epitaxy step.

Figure 3, on the left, shows the cross-sectional SEM images of the porous layers in the as etched state with varying thickness and porosity. The layers are mesoporous according to the definition by IUPAC since the pore sizes are between 2 and 50 nm [17].

The non-uniformity of the etching depth visible for an etching depth of 200 nm is not further increased, but is reduced as the etching proceeds. The etching depth is uniform within ±6% at an etching depth of 1.4 µm. The porosity gradient is not visible in the as etched state.

### 3.3. Stain Etched Porous Silicon Reorganization

The epitaxy process for PSI solar cells based on substrate wafers that feature an electrochemically etched double layer had to be adapted to the stain etched porous Si layer. This was necessary since the new surface part with highest porosity functions not only as a predetermined breaking point but also as a starting layer for epitaxy.

Figure 3, on the right, shows porous Si layers with different thicknesses after the epitaxy process as mentioned above. The variation in porosity becomes most obvious after the epitaxy. The high process temperature causes the reorganization of the pores. Layers of a thickness of 200 nm and 400 nm are characterized by the larger main pores running over the whole layer thickness. At a thickness of 600 nm a porosity profile starts to evolve which is clearly visible at a layer thickness of 800 nm. The pore shape after reorganization is still cylindrical, not spherical. For thicker porous silicon layers of 1 µm and more the pore shape develops from cylindrical to spherical pores. For porous layers of a thickness of 1.4 µm the top part of the porous layer right beneath the epitaxial layer dissolves almost completely and a lift-off becomes possible. Only a few pore walls remain to connect the closed surface with the porous bulk layer beneath.

**Figure 4:**
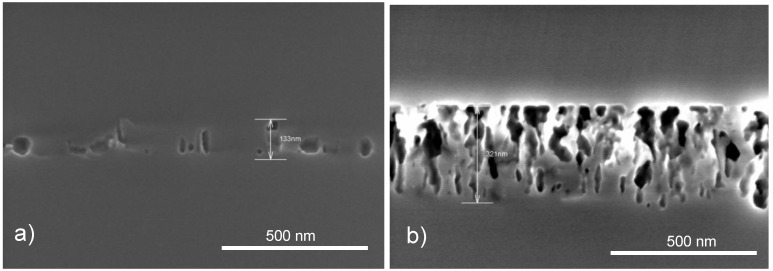
Stain etched porous layers after epitaxy. **(a)** Standard epitaxy process for electrochemically etched samples. The porous layer reorganized to a compact layer with a few voids. Lift-off is not possible; **(b)** Adapted epitaxy process. The porous layer reorganizes to a certain extent and a closed epitaxially grown Si layer results. With this adapted epitaxy process, and a sufficiently thick porous layer, a lift-off is possible.

Figure 4 shows a porous layer after two different epitaxy processes. The sample of Figure 4b underwent the same epitaxy process as the samples shown in Figure 3. The epitaxy process for electrochemically etched samples, the standard process, was applied to the sample depicted in Figure 4a. The reorganization of the layer in case of the standard process leads to a collapse of the porous layer. There are only a few pores left which do not allow a lift-off of the epitaxially grown Si layer. In case of the adapted epitaxy process the porous structure is mainly preserved.

### 3.4. Chemically Etched Substrate of 150 mm Diameter

**Figure 5:**
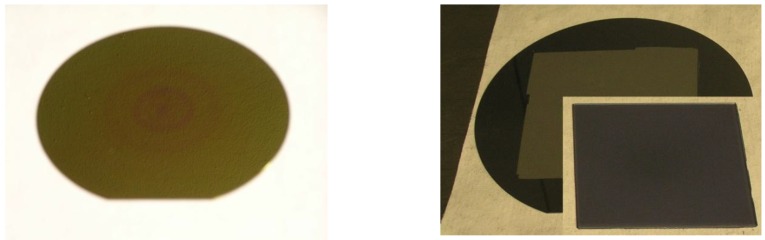
Uniform stain etching of a large area Si wafer (resistivity 2.2 mΩcm). **(a)** The interference color is uniform on the entire wafer surface; **(b)** A Si film of an area 9 × 9 cm^2^ (30 µm thick) is detached from the stain etched 150 mm Si wafer.

Figure 5a shows a chemically etched Si wafer (resistivity 2.2 mΩcm). The surface appears in gold, the color that results from this porosity and thickness of the porous layer. The uniformity of the optical thickness of the pore morphology on the entire 150 mm wafer is obvious from the uniformity in color. In b a detached 30 µm-thick monocrystalline epitaxial Si film with an area of 9 × 9 cm^2^ demonstrates the uniformity of the etching in combination with the subsequent reorganization of the porous layer.

## 4. Discussion

### 4.1. Porosity *versus* Doping Concentration

The range of porosities obtained by changing the resistivity of the substrate wafer from 1.3 to 11.2 mΩcm is 40% (absolute) wide as shown in Figure 1. Even small variations in resistivity between 1.3 and 2.5 mΩcm lead to a difference in porosity of 20% absolute.

The reason for this large impact becomes clear when considering the substrate doping densities instead of specific resistivities. An increase in resistivity from 1.3 to 2.5 mΩcm, *i.e*., a difference of 1.2 mΩcm, corresponds to a change in substrate doping from 8.9 × 10^19^ cm^−3^ to 4.5 × 10^19^ cm^−3^. In contrast, a change in resistivity from 7.7 to 11.2 mΩcm, *i.e*., a difference of 3.5 mΩcm, also corresponds to a decrease of the doping concentration by a factor of about two.

Since all samples show a 600 nm thick porous Si layer the porosity is only determined by the pore wall thickness. This in turn means that the passivation of the pore walls is established at different wall thicknesses corresponding to the doping concentration, *i.e*., the higher the doping concentration the thinner the passivated pore wall.

Therefore the material with resistivity 1.3 mΩcm demonstrates the highest porosity. While the porosity of 56% (1.3 mΩcm) is sufficiently high for layer transfer, 36% (2.5 mΩcm) is critical and the others are definitely too low. But for a successful lift-off, not only the porosity but also the porous layer thickness is relevant as discussed below.

### 4.2. Origin of the Porosity Profile

Besides the doping concentration of the Si wafers also the etch depth controls the porosity. The porosity increases with time due to a thinning of the pore walls in the upper, already etched part of the porous layer. The porosity at the etch front is constant and is determined from the sample of 200 nm etch depth.

The reason for the dissolution of the already formed porous Si in case of the stain etching process is the insufficient pore wall passivation, in contrast to electrochemical etching.

In case of the electrochemical etching process the pore walls of mesoporous Si are passivated by a space charge region effect [18]. The applied voltage induces a space charge region below the substrate surface that is depleted of holes. If the applied voltage and the doping density (above 10^18^ cm^−3^) are large enough, this space charge region becomes sufficiently thin to enable charge carriers (holes) to pass it by band to band tunneling. As long as the pore walls are wider than two times the width of the space charge region the transport of holes from the Si bulk to the surface is possible and the etching of the pore walls proceeds. Alternatively, branching of the pores can occur. The pore walls become depleted of holes if they become so thin that the space charge regions of the adjacent pores begin to overlap. Transport of holes from the Si bulk to the surface of the pores is no longer possible and the etching of the pore walls stops. In highly doped Si the pore diameters are comparable to the width of the space charge region. No overlapping of space charge regions occurs at the pore tips. Therefore the etching proceeds only at the pore tips, and the original wafer surface is preserved. A review on the different models of the porous Si formation process is given e.g., by Smith and Collins as well as Cullis *et al*. [9,10].

In the case of stain etching, the passivation of the pore walls is less stable compared to the electrochemical case because holes are also provided by the oxidizing agent NO_3_^−^ in the etching solution. The chemical reaction transferring electrons from the p-type Si to the electrolyte takes place as long as the band bending leading to an accumulation of electrons at the Si wafer surface and the space charge region exists. Since the concentration of the oxidizing agent in the solution is higher than the minority carrier concentration of electrons in p-type Si the reaction, the band bending and thus the driving force of the dissolution reactions stays constant [12].

According to Kolasinski [15] only the quantum confinement effect as described by Lehman and Gösele [19] can explain the preferential injection of holes into the bottom of the pores at the expense of the walls. That is, holes are injected at a higher rate into the Si bulk compared to the pore walls and thus the etch rate at the bottom of the pores is enhanced. Since there is a non-zero etch rate also at the pore walls the original wafer surface gets eventually dissolved.

### 4.3. Porous Silicon Reorganization

In contrast to the electrochemically etched porous double layer where each layer of the system has only one function, the stain etched highly porous Si surface layer has a double function to serve as a starting layer for the subsequent epitaxy and as a separation layer.

To fulfill both requirements, the surface needs to be closed while the large pores form. It has not been shown in the literature that electrochemically etched porous layers of a porosity as high as used for the separation layer were closed for subsequent epitaxy. For the sample with a resistivity of 2.2 mΩcm an etching depth of 1.4 µm is necessary to generate the suitable porosity profile for a successful lift-off.

The reorganization was described by Müller and Brendel [4]. They simulated the surface closure and the reorganization of the pores within the layer by just assuming the minimization of free surface energy by the jumping of Si atoms to next-neighbor sites. The surface acts as a vacancy sink and thus a thin closed surface layer forms if there is enough Si material available. The silicon is delivered by the pore walls lying deeper in the porous Si bulk. However, if the porosity near the surface is too high, the layer collapses.

Müller and Brendel [4] also stated that for electrochemically formed double layers, the high porosity layer beneath the low porosity layer serves as a sink for vacancies. This means for the stain-etched layers that all vacancies have to move to the front surface since no high porosity layer at the interface to the bulk Si wafer exists. This helps to form a separation layer beneath the closed surface layer as needed for lift-off.

## 5. Conclusions

We demonstrated for the first time that porous Si layers formed by stain etching, allow for a successful layer transfer. A large area epitaxial film was transferred from a Si substrate of 150 mm diameter, which was stain etched to form the double functional porous layer serving as starting layer for epitaxy and as a predetermined breaking point. For a successful lift-off from 2.2 mΩcm substrates an etch depth of at least 1.4 µm was required. This layer thickness was necessary to generate the suitable porosity profile, which allows the surface closure and the formation of the separation layer after the epitaxy process.

The porosity gradient decreases and so does the average porosity for decreasing doping concentration in the range investigated here.

From the electrochemically etched substrates we know that the layers with 20% porosity reorganize and are stable during the standard epitaxy process. Thus the use of the rather lowly doped substrates featuring resistivities between 5 and 12 mΩcm may provide the necessary stability during the standard epitaxy. However, the etching depth has to be large enough to provide enough vacancies which build the separation layer beneath the closed surface.

Even if the deployment of Si substrate wafers with higher resistivity as mentioned above has so far not led to more stable porous layers, the higher substrate resistivities of above 5 mΩcm features as a secondary advantage: Since the dependence of resistivity on doping concentration is less steep for higher resistivities, the parameter windows for etching and epitaxy for successful lift-off will be larger when using higher-resistive substrates. Therefore in a high volume production, where the wafer resistivity specification cannot be as tight as about ±0.1 mΩcm, substrate wafers with such higher resistivity are more suitable.
